# Nationwide Incidence of Acute Alcohol Intoxication in Adolescents in Belgium: A Validation Study of Belgian Health Insurance Data Through Comparison with Retrospective Hospital Chart Data in Antwerp, Belgium

**DOI:** 10.3390/children12020214

**Published:** 2025-02-11

**Authors:** Hanna van Roozendaal, Stijn Verhulst, Inge Glazemakers, Xavier Rygaert, Michiel Callens, Ann De Guchtenaere, Jozef De Dooy, Nico van der Lely, Guido Van Hal

**Affiliations:** 1 Faculty of Medicine and Health Sciences, University of Antwerp, 2610 Antwerp, Belgium; 2 Department of Paediatrics, Antwerp University Hospital, 2650 Edegem, Belgium; 3 University Centre for Child and Adolescent Psychiatry (ZNA-UKJA), 2020 Antwerp, Belgium; 4 Intermutualistic Agency, 1210 Brussels, Belgium; 5 EHSAL Management School, 1000 Brussels, Belgium; 6 Department of Paediatrics, Ghent University Hospital, 9000 Ghent, Belgium; 7 Department of Paediatrics, Reinier de Graaf Hospital, 2625 AD Delft, The Netherlands

**Keywords:** adolescents, minors, alcohol intoxication, population characteristics, health insurance data, hospital chart study, Belgium

## Abstract

**Background/Objectives**: This study investigates the incidence of acute alcohol intoxication (AAI) in adolescents in Belgium, by comparing nationwide estimations based on health insurance data with a recently published hospital chart study in Antwerp. In this way, the scope of AAI among adolescents in Belgium can be estimated more precisely. **Methods**: Health insurance data collected by the Intermutualistic Agency (IMA) regarding 12- to 17-year-olds admitted at emergency departments in Antwerp in 2019–2021 and receiving a blood test to screen for blood alcohol concentration were validated by data derived from a recently conducted retrospective hospital chart study regarding AAI among adolescents in Antwerp. To compare the incidence of adolescents with AAI between these two datasets, a Poisson regression analysis was performed. **Results**: The findings reveal that the approximations based on administrative health insurance data present a significant underestimation of the incidence of AAI (*p* < 0.001): the number of admissions in Antwerp determined by the hospital chart study was 21% higher over 2019–2021. Correcting for this underestimation reveals an estimation for the nationwide average yearly incidence of AAI admissions among adolescents of 33.5 per 10,000 instead of 27.7 per 10,000 in Belgium over 2019–2021. **Conclusions**: These results imply that the occurrence of AAI among Belgian youth is larger than previously estimated. Therefore, a higher fraction of Belgian adolescents are at risk of serious health consequences due to AAI. This underlines the importance of the development of effective intervention strategies regarding indicated prevention and follow-up.

## 1. Introduction

Alcohol use is common among adolescents in Europe. According to the World Health Organisation (WHO), 44% of European citizens between 15 and 19 years old are frequent drinkers [1]. Belgian adolescents show high alcohol consumption rates compared to other European countries, with 53% of Flemish 15-year-olds drinking at least once a month, compared to 37% of international 15-year-olds [2,3]. Although the frequency of alcohol use decreased among Flemish adolescents, the frequency of drunkenness and binge drinking did not decrease [4], with 17% of the 15–16-year-olds binge drinking at least once a month and 30% of the 17–18-year-olds [5]. Binge drinking, usually defined as drinking ≥6 units of alcohol in two hours for boys and ≥4 units in two hours for girls [5] is strongly correlated with a wide range of health risk behaviours [6,7,8]. Most of the adverse health effects of alcohol in teenagers derive from acute alcohol intoxication (AAI), which frequently follows binge drinking [9]. In the short term, AAI is associated with severe complications, such as hypothermia, reduced consciousness, electrolyte disturbances, metabolic acidosis and hypoglycaemia and therefore often requires medical management [10,11]. Adolescents with AAI also have an elevated risk of engaging in delinquent behaviours and using illicit substances in young adulthood [12]. Furthermore, long-term complications of problematic alcohol use in adolescence include an increased risk of alcohol-attributable malignancies, brain damage and alcohol dependency [12,13,14,15,16].

Despite the well-known negative health consequences of AAI, few population-based studies regarding the nationwide incidence of AAI in adolescents have been performed worldwide [17,18,19]. In Belgium, the scale of the problem of AAI in adolescents is not yet identified, for only estimations of incidence numbers of AAI are known. These estimations are made by the Intermutualistic Agency (IMA), which collected administrative data from all seven Belgian health insurance companies. More specifically, since 2008, the IMA has recorded hospital activities of insured patients regarding alcohol intoxication. The collected data include the number of patients who underwent a blood check for alcohol concentration while receiving medical care at an emergency department [20]. However, the IMA does not have diagnostic information or blood test results at its disposal, which means that adolescents with acute alcohol intoxication whose blood alcohol concentration was not tested are not registered by the IMA. Also, patients with a negative result on their blood test are included in the registration of IMA. Therefore, these data can only be used to interpret the number of adolescents with a presumption of AAI. In other words, these data serve as an estimation of the occurrence of AAI in adolescents in Belgium. Regarding the data published online in the IMA atlas, in the year 2018, at least 2200 adolescents (12- to 17-year-olds), or 30 per 10,000 adolescents, were admitted to hospital with a suspicion of AAI in Belgium [21,22].

Recently, a retrospective hospital chart study (Antwerp AAI study) on the incidence of AAI among adolescents in the city of Antwerp, Belgium, has been conducted by the researchers of this article, which probably gives a more accurate approximation of the AAI incidence in this region [23], as the results of alcohol blood tests were taken into account. This Antwerp AAI study showed a mean yearly hospitalisation rate of 31 per 10,000 10–17-year-old inhabitants in the city of Antwerp over the years 2015–2021. In the current study, data from the Antwerp AAI study are used to validate the estimations based on national health insurance data regarding AAI. Therefore, this study aims to map the scale of the problem of AAI in adolescents in Belgium more precisely. This is needed to assess the severity of the situation and highlight the need to develop suitable preventive measures.

## 2. Materials and Methods

### 2.1. Study Design and Outcome Variables

A selection of nationwide health insurance data of the IMA were compared with data derived from a recently conducted retrospective hospital chart study regarding AAI in the city of Antwerp (Antwerp AAI study) [23]. The city of Antwerp is the second largest city in Belgium and has 545,000 inhabitants, of which 40,700 are 12–17-year-olds, in 2024 [24]. The analysed data cover the scope of the city of Antwerp.

The comparison between the IMA data and hospital chart data (Antwerp AAI study) was made for data from the same hospitals in the city of Antwerp over the years 2019–2021. In this way, the nationwide IMA data were validated, and the primary outcome of the incidence of adolescents admitted with AAI in Belgium over 2019–2021 was calculated using extrapolation. Secondary outcomes were (medical) characteristics and trends in incidence rates of the AAI population in both the city of Antwerp (2019–2021) and the whole of Belgium (2008–2021).

### 2.2. Data Collection

IMA collected nationwide data of 12- to 17-year-old insured adolescents admitted at an emergency department in Belgium in the period 2008–2021, who received a blood test to screen for blood alcohol concentration (BAC) during admission, on the same day. Two different IMA datasets were used in this study. The first dataset consisted of hospital-specific incidence numbers of the city of Antwerp throughout 2019–2021, needed for the validation of the incidence of AAI among Belgian adolescents (primary outcome), and was shared by the IMA with the research team for the purpose of this study. This dataset consisted of exact numbers of patients with a presumption of AAI per year per hospital in Antwerp. It included the six hospitals of the Hospital Network Antwerp (ZAS Augustinus, ZAS Vincentius, ZAS Sint-Jozef, ZAS Middelheim, ZAS Jan Palfijn, ZAS Stuivenberg/Cadix) and two hospitals of the Helix network, namely the Antwerp University Hospital (UZA) and AZ Monica. The second dataset was retrieved from the IMA atlas, the online dashboard where the IMA publishes health statistics of insured patients, which is publicly available [25]. This dataset was used for assessing the secondary outcomes. Next to the yearly number of admissions of 12- to 17-year-olds who received a BAC test (in ratio per 10,000 12- to 17-year-olds) from 2008 to 2021, the categorical variables sex and geographical information (based on residence data of the patients on 31 December of the year of admittance) of these patients have been recorded. Furthermore, over the years 2008–2017, data regarding the recurrence rate and day of the week of admissions have also been recorded and extracted for this study.

For assessing the primary outcome of this study, a part of the data collected by the Antwerp AAI study was used [23].

In the Antwerp AAI study, data were derived from hospital charts of 10- to 17-year-old adolescents admitted with AAI in the eight above-mentioned hospitals of the city of Antwerp with a paediatric ward throughout 2015–2021. Selection of hospital charts took place via a positive blood alcohol concentration (BAC, >0.03 or >0.1 g/L, depending on the laboratory) and was complemented via screening of triage logs (only in UZA) and via screening software making use of the search terms ‘intoxication’, ‘alcohol’, ‘ethanol’ and ‘drunkenness’ (only in ZAS Augustinus, ZAS Vincentius and ZAS Sint-Jozef, the former ‘GZA’ hospitals). After screening, all patients with a positive BAC and/or clinically diagnosed alcohol intoxication by the emergency doctor were included in the Antwerp AAI study. In the current study, the following variables from the Antwerp AAI study have been used: the number of 12- to 17-year-old patients admitted in 2019, 2020 and 2021, sex, age, year of admission, day of the week of admittance, blood alcohol concentration (in g/L), urine drug screening (whether performed and result when performed), details of referral to other healthcare workers after admittance, and recurrence rate.

### 2.3. Statistical Analyses

A Poisson regression analysis was performed to analyse the incidence of adolescents with AAI in Belgium in 2019–2021 (primary outcome). This regression model was considered suitable because of the primary outcome being count data. Therefore, a Poisson regression model with 95% confidence intervals (CIs) was used to estimate the difference in incidence between the IMA data and hospital chart data, taking into account potential covariates and effect modifiers. CIs not including one were considered to be statistically significant.

The secondary outcomes of the study were analysed using descriptive statistics, with categorical variables being expressed as proportions and continuous variables as means (standard deviation, SD) or medians [interquartile range (IQR)], depending on the distribution of the variable (determined via histogram assessment and Kolmogorov–Smirnov test performance). The yearly incidence rates of the hospital chart data were calculated by dividing the number of admissions reported in each year by the corresponding number of 12- to 17-year-old inhabitants of the city of Antwerp in January of the same year [24] and expressed per 10,000 12- to 17-year-old inhabitants. On the other hand, the yearly incidence ratios of the health insurance data were presented by the IMA atlas [25], and therefore, the absolute number of yearly admissions was calculated by multiplying the yearly incidence ratio with the corresponding number of inhabitants of Belgium each year divided by 10,000 [26]. In addition, Pearson’s chi-squared tests were used to analyse trends in incidence rates and associations between incidence rates and patient characteristics (the categorical variables sex and residency). Furthermore, Mann–Whitney U tests (for variables with two categories) and Kruskal–Wallis tests (for variables with more than two categories) were performed to analyse associations between demographics and medical characteristics within the hospital chart dataset.

We used an alpha level of 0.05 for all statistical tests. IBM SPSS Statistics for Windows, Version 28.0 (Armonk, NY, USA: IBM Corp), was used for the statistical analyses.

## 3. Results

### 3.1. Comparison of AAI Incidence Between IMA Data and Hospital Chart Data

**Figure 1:**
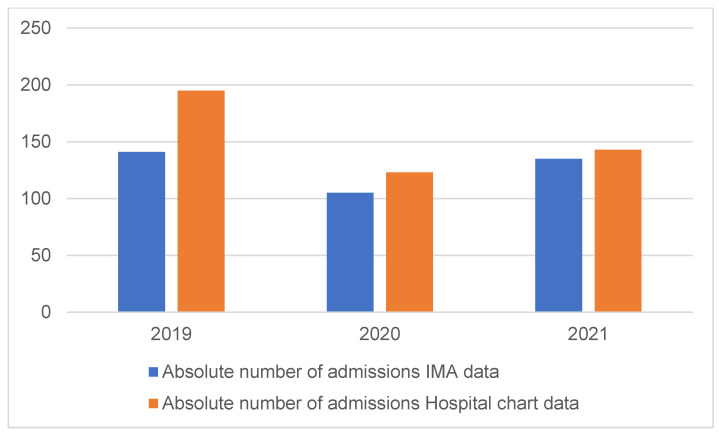
Absolute number of admissions of 12- to 17-year-olds with (suspicion of) acute alcohol intoxication according to IMA data and hospital chart data for the city of Antwerp in 2019, 2020 and 2021.

When examining the absolute incidence of AAI in 12- to 17-year-olds in the eight main hospitals in the city of Antwerp over 2019–2021, we observed a total of 381 admissions registered by IMA and 461 in the hospital chart dataset. This amounts to a mean incidence rate of 35.6 (SD = 6.1) per 10,000 in the IMA data and 43.2 (SD = 11.5) per 10,000 in the hospital chart data. Figure 1 shows the number of admissions of both the IMA data and the hospital chart data separately per year.

Overall, the number of AAI admissions among 12–17-year-olds in Antwerp from 2019 to 2021 was 21% higher when screened via hospital charts, compared to the IMA registration (461/381). Assuming the hospital chart data to be more accurate, we calculated the expected mean yearly incidence rate of 12- to 17-year-olds admitted to hospital with the suspicion of AAI in Belgium throughout 2019–2021, with the above correction taken into account: instead of an average of 27.7 per 10,000 admissions per year, it would be 33.5 per 10,000 per year (29.7 × 1.21). However, caution should be taken with the interpretation of this calculated mean yearly incidence rate, as we cannot assume that the same difference in registration will be prevalent in all regions of Belgium.

A Poisson model was used to analyse the differences in the number of admissions between the two different datasets. In this model, the number of admissions was the dependent variable (count data), and the source of the data (IMA or hospital chart) was the independent variable. The hospital group (ZAS, GZA, UZA or AZ Monica) and the interaction between the hospital group and the data source were also included in the model. The overall model revealed a significant difference between the number of admissions of the IMA dataset and the hospital chart dataset (*p* < 0.001), with both the dependent variables and covariates being significant (*p* = 0.009 for source of the data; *p* < 0.001 for hospital group; *p* < 0.001 for interaction between hospital group and source of the data). However, when running a subgroup Poisson regression per hospital group, only three out of eight hospitals (ZAS Augustinus, ZAS Vincentius and ZAS Sint-Jozef, all former ‘GZA’ hospitals) showed a significant difference (*p* < 0.001). Here, the subgroup analysis showed that the chance of finding an admission in one of these former ‘GZA’ hospitals by the IMA data was 57% lower (CI 0.326–0.576, *p* < 0.001) compared to the hospital chart data. No significant differences were found for the other hospitals within the city of Antwerp. Therefore, the overall difference in the number of admissions between the IMA dataset and hospital chart dataset seemed to be explained by differences within the former ‘GZA’ hospitals.

### 3.2. Characteristics of Adolescents with AAI Based on Health Insurance Data (IMA)

From 2008 to 2021, the median yearly incidence rate of 12- to 17-year-olds admitted to hospital with a suspicion of AAI in Belgium was 30 [29.75–31.25] per 10,000, according to the health insurance data of the IMA. When looking at absolute yearly admissions, this amounts to a median of 2250 (2212–2365) ED admissions due to AAI per year in Belgium. Over the years, no significant change in yearly incidence rates was seen (*p* = 0.996). Table 1 shows both the incidence rate per 10,000 and the absolute number of admissions per year in Belgium.

The median yearly incidence rate did not differ significantly between males and females (*p* = 0.701): over 2008–2021, it was 31.5 [29.75–32.0] per 10,000 for males and 29.0 [26.75–32.0] per 10,000 for females. Furthermore, when analysing the incidence rate per year for males and females separately, no significant trends over the years were observed (*p* = 0.890 for males; *p* = 0.994 for females).

Regarding geographical information, significant differences were seen in mean incidence rates per year between the three regions of Belgium (*p* = 0.025), with Brussels having the lowest mean incidence rate of 17 per 10,000, followed by Flanders (27 per 10,000) and Wallonia (37 per 10,000). Furthermore, the mean incidence rate over 2008–2021 also differed significantly on the province level (*p* < 0.001), with the highest rate in Luxembourg and the lowest rate in Brussels, as shown in Table 2. When focussing on the province of Antwerp specifically, a median incidence rate of 27.5 [20.75–28] per 10,000 over 2008–2021 was seen, with no significant changes over the years. The province of Antwerp shows a lower mean incidence rate compared to the average, as presented in Table 2.

In addition, the IMA data showed a recurrence rate of 3.5% throughout 2008–2017, which corresponds to the proportion of insured persons having more than one admission with a suspicion of AAI per year. Furthermore, the data show that 60% of the 12- to 17-year-olds with a suspicion of AAI between 2008 and 2017 were admitted during the weekends (Friday to Sunday).

### 3.3. Characteristics of Adolescents with AAI Based on Hospital Chart Data (Antwerp AAI Study)

Over the years 2019 to 2021, on average, 154 12- to 17-year-olds (37.17 per 10,000) were admitted with AAI in the city of Antwerp every year, which corresponds to a mean incidence rate of 43 (SD = 11.53) per 10,000. Significant differences were seen in incidence rates per 10,000 over the years 2019–2021 (*p* = 0.045). Here, the highest incidence was seen in 2019 (195 admissions or 56.2 per 10,000) and the lowest in 2020 (123 admissions or 34.4 per 10,000), as shown in Table 3. Table 3 also shows the main baseline characteristics of all 461 admitted adolescents in the city of Antwerp with AAI in 2019–2021.

As shown in Table 3, the overall admission rate of males and females was equal (*p* = 0.244), with 52.7% of patients being males and 47.3% being females. The median age of the patients was 16.5 [15.5–17.3] years old. Noticeably, females were significantly younger at the time of admission than males (*p* < 0.001), with females having a median age of 16.3 [15.3–17.0] years old and males 16.7 [15.7–17.4] years old.

Regarding the medical characteristics of the admitted patients, a median BAC at the time of admission of 1.88 [1.20–2.30] g/L was seen, which was tested in 80.0% of the cases. This corresponds to around 6 to 10 alcoholic drinks, not taking into account the delay in measuring BAC after the last drink. No significant differences were observed between males and females regarding BAC (*p* = 0.395). However, when looking at age, a significant difference was noticed (*p* = 0.014): the older the patients, the higher the median BAC. In addition, a breathalyser was used for the detection of alcohol in 19 of the 157 cases (12.1%) in the former GZA hospitals with a median measured alcohol concentration of 1.26 [1.10–1.63] g/L. Furthermore, in 31.7% of the cases, a urine drug screening was performed (146/461). In 15.6% of these patients, there was a positive drug screening for at least one drug, of which cannabis was the most commonly used. In addition, a recurrence rate of 0.9% over 2019–2021 was seen; however, only the recurrent admissions within the same hospital were registered. Moreover, most patients with AAI (65.7%) were admitted to the hospital during the weekends (Friday to Sunday). Finally, the data show that most of the patients did not receive additional care after their emergency admission (73%).

## 4. Discussion

This study analysed the incidence of AAI in adolescents in Belgium, by comparing nationwide estimations of health insurance data collected via the IMA with the assumably more accurate data of the city of Antwerp in Belgium, which were recently collected by a retrospective hospital chart study (Antwerp AAI study) [23]. The results of the study imply the incidence of AAI among 12–17-year-olds to be higher than the estimations of the IMA: the number of admissions in the city of Antwerp was 21% higher over 2019–2021. Extrapolating this insight to the nationwide context leads to an estimation of an average yearly incidence of AAI admissions of 33.5 per 10,000 instead of 27.7 per 10,000 in Belgium over 2019–2021. However, this projection of the retrospectively obtained data from the city of Antwerp to the whole of Belgium should be interpreted with caution.

There might be explanations for the differences between the analysed datasets. Primarily, the Antwerp AAI study did not collect cases solely on BAC results, but also on triage logs and/or search terms within notes of healthcare workers. Only in 80% of the hospital chart cases was a BAC screening performed. The main objective was to also include patients with AAI who did not undergo BAC screening. The relative fraction of additional patients who did not undergo BAC screening (i.e., 20%), is comparable with the relative underestimation of the administrative data (i.e., 21%). Also, in the Netherlands, where AAI cases are collected through registration by paediatricians, a BAC screening is not always performed. In a comparable study, 12% of Dutch AAI cases did not undergo a BAC screening [17]. Secondly, different criteria were used in the two datasets to demarcate the inclusion of patients. In the hospital chart data (Antwerp AAI study), patients who were admitted to another department (e.g., paediatrics) besides the emergency department, or patients who did not stay for at least one night, were also included. This is different in the IMA database. Here, patients were only included when, next to undergoing a BAC screening, they were admitted to either the emergency department or another department in the hospital for at least one night. Also, patients who received their BAC screening on another day than the admission were missed by IMA. In this context, IMA estimated the amount of 12- to 17-year-old patients undergoing a BAC screening who did not meet the other criteria for inclusion, around 17% [20]. Lastly, the IMA data did not include uninsured AAI patients. Almost 99% of the Belgian population is health-insured [20] and therefore the uninsured group is negligibly small at first sight. But we cannot rule out the possibility that on the hospitalisation level, the uninsured group is disproportionally represented. In contrast, uninsured patients were included in the Antwerp AAI study. Altogether, the above explanations could have led to selection bias, and therefore to a less accurate measurement of the incidence of AAI in adolescents by the health insurance data compared to the hospital chart data.

Hence, because of the above-mentioned reasons, we carefully assume the calculated yearly incidence of 33.5 per 10,000 to be more accurate. Nevertheless, possibly this is still an underestimation. There are relevant considerations in this regard. First, possibly the hospital chart data are incomplete due to unreported cases. For example, some adolescents with AAI might have visited a general practitioner instead of an emergency department or might have stayed at home under the supervision of a parent. Therefore, also the hospital chart data alone might be an underestimation of the total incidence of AAI in adolescents in the city of Antwerp. Furthermore, the minimum age in both the datasets was set at 12 years old, albeit we have seen cases of AAI in younger patients. In the Antwerp AAI study, hospital charts of 10- to 17-year-olds were screened, and the youngest patients were 11 years old; seven 11-year-old patients were admitted to hospitals in Antwerp in 2019–2021 [23]. The underestimation would have been 23% instead of 21% if these patients would have been included in the comparison. In addition, the Poisson model showed that only the former GZA hospitals were accountable for the difference in incidence between the two datasets. A possible explanation could be that only in these hospitals the selection of patients was not performed by positive BAC screening only, but complemented by screening software making use of alcohol-specific search terms. Unfortunately, this software was not available at the other hospitals at the time of data collection. Nonetheless, it could be assumed that using similar software in the other hospitals, would have led to a higher overall incidence. However, here it should be taken into account that some of the adolescents were included based on a clinical diagnosis only. Although a clinical diagnosis by emergency doctors based on the patient’s and/or caregiver’s history and characteristic physical findings is typically sufficient, BAC assessment confirms the diagnosis [27]. Finally, the lockdown in 2020 due to COVID-19 is another debatable matter in considering a possible underestimation of the average yearly incidence rate. Possibly the various lockdown measures have led to the lower incidence found in 2020, although non-significant. A significant decrease in AAI incidence among adolescents during COVID-19 was demonstrated by researchers in the Netherlands and Italy [28,29]. However, it should be noted that both datasets would have shown a relative decrease in incidence and therefore, the influence of COVID-19 on our primary outcome is possibly insignificant.

However, an overestimation of the incidence ratio of the hospital chart data compared to the administrative data should also be considered, due to the inclusion of patients admitted to Antwerp-based hospitals who were not residents of the city of Antwerp in the Antwerp AAI study. This is especially relevant for the Antwerp University Hospital, as this hospital is located on the outskirts of Antwerp. A limitation of this study in this regard is that the domicile of the patients was not taken into account. 

The projection of data differences between the IMA and hospital chart data from the Antwerp region to the general Belgian population should be interpreted with caution, because of potential contextual differences among Belgian regions. For instance, potential differences in drinking cultures might exist between different provinces in Belgium. Also, differences in population density per province could be of influence, as previous research showed that adolescents from rural areas are at higher risk of alcohol use [30]. In addition, religion also influences alcohol use among adolescents [31] and differences in religious affiliation among adolescents from different provinces might exist. Therefore, the findings from the urban area of Antwerp might not be suitable to be extrapolated to other urban areas with cultural differences or rural areas of Belgium. Secondly, there might be substantial differences in the medical management of AAI between provinces, affecting the accuracy of the health insurance data. For instance, paediatricians and emergency room doctors who have a lower threshold for testing on BAC in one province, or hospitalisation being more accessible for adolescents with AAI in another province. The above-mentioned differences between the (urban) context of Antwerp and other provinces of Belgium were not taken into account in this study. It is highly debatable whether the joint effect of these differences would significantly influence the findings, and if so, how the results should be corrected.

Comparing the newly calculated incidence rate of 33.5 per 10,000 12–17-year-olds in Belgium to incidence rates found in other European countries, the following can be concluded. It is substantially higher than the incidence rate in the Netherlands (26 per 10,000 10–17-year-olds) [17,32] but about three times lower than the incidence rate in Wales (99 per 10,000 10–17-year-olds) [18]. The above differences could be explained by the drinking patterns of 15-year-olds in these countries, captured by the Health Behaviour in School-aged Children (HBSC) study in 2010 [33]. This survey showed a higher weekly alcohol consumption rate in Wales compared to Belgium and the Netherlands (29.5% in Wales, 23.3% in Belgian Flanders, 22.3% in Belgian Wallonia and 20.4% in the Netherlands). Also, a higher drunkenness level was seen there: 48.6% of 15-year-olds in Wales have been drunk two or more times in their lifetime, compared to 27.5% Belgian Flanders, 24.5% in Belgian Wallonia and 18.1% in the Netherlands. Comparison with other European regions is impeded due to the absence of published nationwide incidence rates of AAI among adolescents.

Regarding the secondary outcomes, the results of this study support the hypothesis that, although regular drinking declined in Belgian adolescents over the past decade, binge drinking did not [4]: in both the IMA and the hospital chart data no decreasing trend in AAI has been found. In addition, more similarities were found between the two different methods. First, an equal distribution of both males and females was seen in both methods, comparable with a similar study in Spain [34]. Furthermore, both methods showed that the majority of cases were admitted during the weekends. However, the recurrence rate differed among the different methods: 0.9% in the Antwerp AAI study and 3.5% in the IMA data. Here, the recurrence rate of the hospital chart data might have been an underestimation due to the anonymity of patients between the various hospitals. The recurrence rate of IMA might be more accurate since similar rates have been found in previous research [34]. Other variables, such as distribution of age among sex, BAC results and drug screening could not be compared, due to unavailable data from IMA.

Although this study is the first to address a validation of nationwide estimations of AAI incidence in Belgium, we hypothesise that there are still reasons to assume that the incidence found in this study is not completely accurate, as set out earlier. Further research is crucial to gain more insight into nationwide incidence ratios. More precise estimations could be obtained by creating a nationwide registration system used by emergency doctors all over the country to keep track of adolescent patients with AAI—a similar registration system to the one currently in place in the Netherlands [17].

The results of the study imply that the occurrence of AAI in adolescents in Belgium might be greater than previously estimated by health insurance data. In other words, it seems a larger fraction of Belgian adolescents have problematic alcohol intake with potentially serious health consequences than previously thought. Therefore, the demand for effective care is higher than previously assumed. Hence, effective intervention strategies regarding the prevention of AAI and the follow-up of AAI patients are more relevant than ever before. This is consistent with the view of the Belgian federal government, which recently published a new alcohol plan, containing both preventive measures regarding marketing of alcohol and alcohol availability, and recommendations regarding follow-up interventions for adolescents with AAI, amongst others [35]. This latter recommendation is also in line with various previous research, advising the development of a screening and intervention programme following alcohol intoxication in adolescents, to prevent adverse health and social consequences resulting from this behaviour [9,12,34]. For this reason, an outpatient clinic for screening, intervention and follow-up of adolescents admitted with AAI has recently been set up by the researchers of this study in the city of Antwerp. Family treatment and motivational enhancement therapy are also incorporated within this follow-up setting. When pilot tests of the outpatient clinic show promising results, it is the intention to set up a national network of these outpatient clinics in Belgium with the help of the federal government.

## 5. Conclusions

This study showed a significant difference in the estimations of AAI among adolescents based on Belgian health insurance data compared with the incidence of AAI based on a retrospective hospital chart study from patients in the city of Antwerp [23]. This latter method showed a 21% higher incidence over 2019–2021. When projecting this difference in incidence to the entire Belgian population, a nationwide average yearly incidence over 2019–2021 of 33.5 per 10,000 adolescents (12- to 17-year-olds), instead of 27.7 per 10,000, was approximated. These results should be used with caution because the calculated incidence is possibly still a misrepresentation and the regional differences across Belgium most likely do not allow for uniform extrapolation. Nevertheless, we conclude the occurrence of AAI among Belgian youth is bigger than previously estimated and therefore more Belgian adolescents are at risk of serious health consequences due to AAI. This underlines the importance of the development of effective intervention strategies regarding indicated prevention and follow-up for AAI among adolescents in Belgium.

## Figures and Tables

**Table 1:** Estimations of AAI incidence rate per 10,000 12- to 17-year-olds and absolute number of admissions per year, according to health insurance data.

Year	Incidence Rate	Number of Admissions
2008	28	2132
2009	29	2191
2010	30	2246
2011	30	2229
2012	30	2221
2013	31	2302
2014	32	2378
2015	31	2295
2016	30	2219
2017	32	2381
2018	30	2253
2019	32	2430
2020	21	1620
2021	30	2361

**Table 2:** Mean incidence rate per 10,000 12- to 17-year-olds per province over 2008–2021, according to health insurance data.

Province	Mean Incidence Rate
Antwerp	25
Brussels	17
Hainaut	41
Limburg	19
Liege	32
Luxembourg	48
Namur	41
East Flanders	28
Flemish Brabant	24
Walloon Brabant	30
West Flanders	39
Unknown	28

**Table 3:** Baseline characteristics of 461 12- to 17-year-old patients admitted with AAI between 2019 and 2021 in Antwerp, according to hospital chart data.

Characteristics	Total
	*n* = 461
Year of admission, *n* (rate per 10,000)	
2019	195 (56.2)
2020	123 (34.4)
2021	143 (38.9)
Sex, *n* (%)	
Male	243 (52.7)
Female	218 (47.3)
Age (years)	
Median [IQR]	16.5 [15.5–17.3]
Range	12.1–17.9
Blood alcohol concentration (g/L) (*n* = 369)	
Median [IQR]	1.88 [1.20–2.30]
Range	0.03–3.95
Urine drug screening (*n* = 146), *n* (%)	
Negative	123 (84.4)
Positive for cannabis	13 (9.2)
Positive for cocaine	1 (0.7)
Positive for Ecstasy (XTC)/amphetamines	7 (5.0)
Positive for benzodiazepines	7 (5.0)
Referral after admittance (*n* = 377), *n* (%)	
None	273 (72.4)
Paediatrician	12 (3.2)
General practitioner	20 (5.3)
Psychosocial assistance	15 (4.0)
Child and adolescent psychiatry	31 (8.2)
Addiction care	3 (0.8)
Day of admittance, *n* (%)	
Monday–Thursday	158 (34.3)
Friday-Sunday	303 (65.7)
Recurrence, *n* (%)	
Recurrent admissions	4 (0.9)

## Data Availability

The national IMA dataset is publicly available from https://atlas.ima-aim.be/databanken (accessed on 10 August 2023). The other datasets used in this study are available from the corresponding author upon reasonable request. The data are not publicly available due to legal issues, as data transfer agreements were drawn up between the collaborating hospitals in which the public availability of data was not described.

## Abbreviations

The following abbreviations are used in this manuscript:

AAI	Acute alcohol intoxication
IMA	Intermutualistic Agency
WHO	World Health Organisation
BAC	Blood alcohol concentration
ZAS	Hospital Network Antwerp
UZA	Antwerp University Hospital
SD	Standard deviation
IQR	Interquartile range
CI	Confidence Interval
